# Challenges in implementing pharmacist-led admission medication reconciliation in hospital practice: a qualitative study

**DOI:** 10.1186/s12913-025-13869-1

**Published:** 2025-12-12

**Authors:** Kuan-Lin Chen, Ming-Fang Wen, Hsin-Yu Tsai, Jo-Hsi Chen, Yen-Ming Huang

**Affiliations:** 1 Department of Pharmacy, National Taiwan University Hospital, Taipei City, 100229 Taiwan; 2 Department of Pharmacy, Taipei City Hospital Zhongxing Branch, Taipei City, 103212 Taiwan; 3 Graduate Institute of Clinical Pharmacy, College of Medicine, National Taiwan University, Taipei City, 100025 Taiwan; 4 School of Pharmacy, College of Medicine, National Taiwan University, Taipei City, 100025 Taiwan

**Keywords:** Implementation, Medication reconciliation, Pharmaceutical service, PRECEDE-PROCEED, Qualitative, Workflow

## Abstract

**Background:**

Pharmacist-led admission medication reconciliation (MedRec) plays a critical role in optimizing pharmacotherapy during care transitions. Despite its benefits are well established, real-world implementation remains limited. To address this gap, this study explored context-specific barriers and facilitators affecting admission MedRec implementation in a hospital setting.

**Methods:**

This narrative qualitative study used semi-structured interviews to explore factors influencing the implementation of pharmacist-led admission MedRec at a medical center in Taiwan. Fifteen pharmacists with admission MedRec experience and varying seniority levels were purposively recruited for face-to-face interviews. The interview guide was developed using the PRECEDE-PROCEED model and covered predisposing, enabling, and reinforcing factors, as well as physical and social environmental influences. All interviews were audio-recorded, transcribed verbatim, and analyzed using inductive thematic analysis. Although coding was conducted inductively, the resulting themes were subsequently organized within the PRECEDE-PROCEED framework.

**Results:**

Five overarching themes were identified. Predisposing factors reflected pharmacists’ positive views of admission MedRec but noted challenges with prioritizing it in daily practice. Reinforcing factors pointed to a lack of performance feedback and limited external recognition. Enabling factors highlighted gaps in professional competencies and insufficient training opportunities. Physical and social environmental barriers, including limited information system support, frequent workflow interruptions, and unclear institutional norms, further impeded implementation.

**Conclusions:**

Major barriers to implementing pharmacist-led admission MedRec included time constraints, unclear prioritization, and inadequate feedback mechanisms. While pharmacists recognized the value of admission MedRec, it was frequently deprioritized during routine practice. Using the PRECEDE-PROCEED model facilitated a structured assessment of individual and contextual determinants. These findings provide practical direction for developing targeted strategies to support and sustain admission MedRec implementation in hospital settings.

## Background

Medication reconciliation (MedRec) refers to a structured and systematic process for verifying a patient’s medication history, aligning it with current treatment plans, and resolving unintended medication discrepancies. It is a vital intervention for reducing medication errors during transitions of care [1]. Within the broader context of pharmaceutical care, MedRec serves as a core component of seamless care, an approach designed to maintain continuity and safety as patients move across healthcare settings [2, 3]. Evidence shows that nearly 6% of hospitalized patients experience medication errors [4], more than half of which occur during care transitions [5]. These errors often stem from medication discrepancies, which are defined as unexplained differences between a patient’s medication regimens across different stages of care [6], and they may lead to patient harm [7].

In 2017, the National Taiwan University Hospital launched a pharmacist-led admission MedRec service to enhance inpatient medication safety. Pharmacists are particularly effective in identifying medication discrepancies, as their medication histories are more accurate and comprehensive than those obtained by other healthcare professionals [8, 9]. The service was first piloted in one ward with favorable outcomes, including improved detection of medication errors and better interprofessional collaboration [10, 11]. Table 1 outlines the admission MedRec process developed based on this pilot. Despite these early successes, the hospital-wide adoption has remained limited, with relatively few admission MedRec services being carried out by pharmacists.

**Table 1:** Hospital inpatient admission medication reconciliation process

Steps	Actions
Patient admission	• Obtain the patient’s written consent to access the PharmCloud medication records. This step is performed by nurses or physicians upon the patient’s admission to the ward.
Retrieving cloud medication record	• The pharmacist accesses the patient’s three-month medication history from the electronic medical record (EMR) system, with data batch-downloaded from the PharmCloud system the day after consent is obtained. • The pharmacist reviews the records to identify medications that the patient is currently using. • The pharmacist confirms the details of current medication use with the patient or caregiver (optional).
Creating best possible medication history (BPMH)	• The pharmacist compiles a comprehensive and accurate list of the patient’s current medications.
Comparing medication lists	• The pharmacist compares the BPMH with the patient’s admission medication orders to identify discrepancies.
Reconciling discrepancies	• If unintended medication discrepancies are identified, the pharmacist discusses with the physician to determine whether changes to the admission medication orders are needed.
Documentation	• The pharmacist enters the BPMH in the EMR system. • The pharmacist documents the admission medication reconciliation process in the EMR system.

Since 2014, MedRec has been designated as a patient safety goal for hospitals in Taiwan [12], and this requirement remains in effect as of 2025. To support this initiative, the National Health Insurance (NHI) Administration launched the PharmCloud system in 2013, which allows healthcare providers to access patients’ medication histories across institutions. Several hospitals have incorporated PharmCloud into MedRec processes, with studies showing potential benefits [13–15]. Nevertheless, most investigations have been small-scale and confined to specific wards rather than system-wide implementation. Roles and responsibility for MedRec also vary, with some hospitals assigning it to pharmacists and others to physicians or nurses. Unlike countries, such as Slovenia, where pharmacist-led MedRec is nationally established and reimbursed [3], Taiwan’s NHI does not reimburse MedRec, and the Pharmacist Act offers only a broad definition of pharmaceutical care without specifically identifying MedRec as a pharmacist’s mandated responsibility. This contributes to slow institutional uptake, leaving MedRec largely in the pilot or early implementation stage in many hospitals. A common challenge in healthcare innovation is the lengthy lag between research and routine practice. Estimates suggest that even effective interventions can take up to 17 years to be fully integrated into standard clinical workflows [16]. The process of MedRec faces similar obstacles, as its implementation is often resource-intensive and operationally complex [17, 18]. Many healthcare institutions report being able to provide MedRec services to only a limited number of patients [19]. These challenges underscore the importance of understanding the factors that hinder its broader implementation. Implementation science, defined as the study of strategies to promote the uptake of evidence-based practices, offers a structured approach to addressing this gap [20]. By exploring contextual determinants and informing targeted implementation strategies, it helps bridge the divide between research and routine care [21]. Local context is particularly crucial, as barriers, facilitators, and resource differ between institutions. Site-specific qualitative insights are therefore essential for understanding nuances in workflow, organizational culture, and professional roles that broader studies may overlook. Despite this need, limited qualitative research has explored the factors that influence hospital pharmacists’ implementation of admission MedRec in Taiwan. This gap has restricted understanding of how system-level policies intersect with the day-to-day realities of pharmacy practices at the institutional level. 

This study used a qualitative approach to exploring the underlying factors affecting the implementation of pharmacist-led admission MedRec in a hospital setting. Our objective was to generate in-depth, context-specific insights into practical challenges and facilitators encountered by pharmacists in real-world practice. The findings are intended to inform the development of local implementation strategies and provide transferable lessons for other hospitals or institutions facing similar challenges.

## Methods

### Study design

This narrative study explored factors influencing the implementation of pharmacist-led admission MedRec within a hospital setting. The rationale for using a qualitative approach was to first generate in-depth and context-specific insights through interviews, which would later inform the development of a quantitative instrument for further evaluation of the relative importance of the identified factors. Pharmacists with experience performing admission MedRec at the National Taiwan University Hospital were invited to participate in semi-structured interviews. This format allowed participants to express their thoughts freely while enabling interviewers to probe relevant issues in depth.

### Sampling technique

Purposive sampling was used to recruit pharmacists with experience in admission MedRec. To capture a range of perspectives across professional experience, at least three pharmacists from each job tenure category, less than two years, two to ten years, and more than ten years were included. Recruitment continued until data saturation was achieved, defined as the point at which no new themes or insights emerged [22]. Each of the three researchers (KLC, PhD, male; HYT, BS, female; JHC, BS, female), who was responsible for a specific job tenure group, independently assessed when saturation was achieved during coding when no additional themes was identified.

### Interview guide and procedures

The interview guide was based on to the PRECEDE-PROCEED model [23], a behavior-focused framework widely used in healthcare interventions, such as antibiotic prescribing [24] and self-care behaviors [25]. This model was selected because, in the absence of mandatory institutional requirements, pharmacists’ engagement in admission MedRec is largely influenced by individual behavioral determinants, making a behavior-oriented framework particularly appropriate. The PRECEDE-PROCEED model consists of multiple phases, including assessment, implementation, and evaluation. It begins with the identification of individual and environmental factors affecting a target behavior, followed by the design of tailored strategies and evaluation of their effectiveness. In this study, we focused on the assessment phase, which provides a useful structure for categorizing behavioral determinants in qualitative research. Accordingly, the interview questions were organized around five key dimensions: predisposing (e.g., knowledge, beliefs), enabling (e.g., resources, skills), reinforcing (e.g., feedback, rewards), along with the physical and social environments (e.g., infrastructure, organizational culture) that influence admission MedRec implementation. To ensure clarity and relevance, the guide was pilot-tested with two pharmacists who had prior experience conducting admission MedRec and at least two years of post-graduate training. Their familiarity with admission MedRec practices enabled them to provide informed feedback on the content and structure. Data from these pilot interviews were excluded from the final analysis.

Face-to-face interviews were conducted by three hospital pharmacists (KLC, HYT, and JHC). One interviewer (KLC) had nearly ten years of professional experience, while the other two (HYT and JHC) had completed approximately two years of service. To promote rapport and facilitate open communication, interviewers were paired with participants of similar seniority. The most experienced researcher interviewed participants with over ten years of experience, while the others interviewed those with fewer years of service. This approach was intended to enhance mutual understanding through shared clinical backgrounds. Potential participants were invited in person by the interviewers, and all accepted. Each interview lasted around 30–60 min and occurred in a private meeting room or a location where the interviewee preferred and felt comfortable. With participants’ consent, interviews were audio-recorded to ensure accurate capture of the conversation. Participants were encouraged to elaborate on their responses and provide real-life examples when relevant. Regular discussions among researchers were held throughout data collection to ensure consistency in recruitment and interviewing practices.

### Analytical methods

All interviews were transcribed verbatim by a member of the research team. Transcripts were then analyzed using inductive thematic analysis, following the six-phase process outlined by Braun and Clarke [26]. Two researchers (KLC and YMH, PhD, male) independently read each transcript multiple times to familiarize themselves with the data and then generated initial coding using a combination of descriptive and in vivo coding. Any discrepancies in coding were discussed and resolved through regular meetings. One researcher subsequently organized the codes into preliminary themes. At this stage, the five dimensions of the PRECEDE-PROCEED model were used as an organizing framework to structure the emerging themes. The themes were then reviewed and refined through iterative discussions between the two researchers until consensus was reached on the final definitions and thematic structure. Data management, coding, and analysis were performed using MAXQDA 2024 software.

## Results

A total of 15 pharmacists were invited, all of whom agreed to participate. The participants included six (40%) with less than two years of experience, five (33%) with two and ten years, and four (27%) with more than ten years. The average interview duration was 43.8 min, ranging from 19 to 63 min. Participants characteristics are presented in Table 2. The main themes identified from the analysis are summarized below, with illustrative quotes provided in Table 3.

**Table 2:** Demographic and professional characteristics of the interviewees

Assigned Code	Sex	Age (years)	Work experience (years)	Professional degree	Interview duration (mins)
A	male	24	< 2	BPharm	72
B	female	27	< 2	PhramD	28
C	female	27	< 2	PharmD	49
D	female	26	< 2	PhramD	52
E	female	28	< 2	BPharm	49
F	female	24	< 2	BPharm	61
G	female	42	> 10	BPharm	19
H	male	44	> 10	BPharm	20
I	female	33	> 10	BPharm	21
J	female	34	> 10	BPharm	63
K	female	30	> 2 but < 10	MS	32
L	female	28	> 2 but < 10	PhramD	38
M	male	29	> 2 but < 10	PhramD	43
N	female	27	> 2 but < 10	PhramD	47
O	female	29	> 2 but < 10	MS	63

BPharm: Bachelor of science in pharmacy; PharmD: Doctor of pharmacy; MS: Master of science

**Table 3:** Themes and representative quotes on factors affecting pharmacists’ implementation of medication reconciliation

Types of factors		Supporting Quotes
**Predisposing factors**	**Perceptions of admission MedRec**	
	(+) Pharmacists believe that performing admission MedRec can enhance medication safety	“I think it’s pretty important, because doctors often have many patients to take care of, and sometimes patients aren’t really sure about the meds they’re taking. We use their medication history and our drug knowledge to provide suggestions… it’s kind of like helping make the whole care process during the hospital stay more complete.” (Pharmacist B)
	(+) Pharmacists believe that performing admission MedRec can positively support their career development	“From a pharmacist’s point of view, it’s definitely a plus: you really get the chance to use your professional skills. It’s not just about checking inpatient prescriptions and dispensing. With admission MedRec, you actually get to learn a lot along the way.” (Pharmacist I)
	(+) Pharmacists believe that performing admission MedRec can increase awareness of the pharmacist’s role among healthcare professionals and patients	“One of the benefits of doing admission MedRec is that it can shift how other healthcare workers or even patients see pharmacists. People may start to understand our role better and be more willing to involve us in their care.” (Pharmacist A)
	(-) Some pharmacists believe that only certain patients require admission MedRec	“I don’t think every patient needs admission MedRec, but maybe we could focus on certain wards or patients who stay longer. Their situation might change more during the stay, so it would probably help them more.” (Pharmacist F)
	(-) Some pharmacists feel that conducting admission MedRec is too time-consuming	“I’m not really into writing admission MedRec notes because it takes a lot of time; you have to look things up, check the patient’s lab data, and things like that. It’s pretty time-consuming. And when I think about the time it takes versus how often the regimen actually gets changed, I’d rather just use a simpler approach, like calling the doctor directly to talk things through.” (Pharmacist E)
	**Perceived value of admission MedRec**	
	(-) Pharmacists may not fully understand the purpose of completing the full admission MedRec process	“I’m not really sure why we have to write up an admission MedRec note. If the patient’s meds look fine to me, then… I kind of feel like that’s enough” (Pharmacist L)
	(-) Some pharmacists view admission MedRec as a non-essential service or consider it a lower priority	“Compared to admission MedRec, I would rather focus on addressing the patient’s acute issues first. I mean, admission MedRec is still part of our work. It’s not that I don’t want to do it; I just don’t see it as a top priority.” (Pharmacist G)
**Reinforcing factors**	**Performance feedback**	
	(-) Pharmacists feel that failing to sign a pharmacist’s service note carries harsher consequences than not writing it at all	“I mostly just worry that I’ll forget to do the electronic signature and lose part of my bonus! It’s happened to a bunch of my coworkers. They put in all that effort to write the notes but forgot to sign, and still had money deducted. When things work that way, it’s so frustrating that you don’t even feel like writing the notes anymore.” (Pharmacist B)
	(-) Pharmacists believe that the time invested in admission MedRec often yields limited or disproportionate returns	“Um, I don’t think there’s any clear bonus or incentive for doing extra admission MedRec right now. If there were something officially laid out, like get rewarded for doing more, I’d definitely be more motivated. But since it’s not clear, or at least I have no idea what it is, it’s kind of hard to stay motivated.” (Pharmacist M)
	**Community feedback**	
	(+) Pharmacists receive positive feedback from patients when carrying out admission MedRec	“When you do an admission MedRec interview, patients start to realize that pharmacists are actually involved in checking their meds too. They begin to see what we do, and sometimes they’ll even say something nice or give a compliment.” (Pharmacist G)
	(+) Pharmacists receive positive feedback from supervisors for their work of admission MedRec	“If supervisors recognize your work or are willing to arrange more training sessions, it can really help you get more engaged in admission MedRec more quickly.” (Pharmacist M)
	(+) Pharmacists receive positive feedback from other healthcare professionals when conducting admission MedRec	“If admission MedRec makes me feel like I’m part of the team, and if other healthcare professionals genuinely value the pharmacist’s role, then I’d be more willing to do it.” (Pharmacist E)
	(+) Pharmacists experience a sense of accomplishment when completing admission MedRec	“Doing admission MedRec can be a bit of a pain, but when you catch something important, it feels really good, like a little win that gives you a boost.” (Pharmacist L)
**Enabling factors**	**Professional competence**	
	(+) Pharmacists’ ability to assess the appropriateness of a patient’s medications	“You really need to have a good understanding of most medications, like what they’re usually used for, what kind of diagnosis would lead to that prescription, and at least a basic idea of the dose and how it’s taken. That way, when you’re doing admission MedRec, you’re less likely to miss something obvious. Sometimes the problems are right there, but you still need that general sense of what each drug is commonly used for.” (Pharmacist N)
	(+) Pharmacists’ ability to collect relevant patient information	“Since we’re getting info from the hospital system, we need to know our way around it. And when we’re reading through a patient’s chart, we have to know how to pick out the important details. Otherwise, it’s hard to get what we need for admission MedRec.” (Pharmacist A)
	(+) Pharmacists’ ability to document service notes	“During our training, we didn’t really get much guidance on how to write a proper note. There’s not a lot of structured instruction on how to put together a clear, thorough service record.” (Pharmacist C)
	(+) Pharmacists’ communication skills	“Lately I’ve been trying to improve my communication. When I call a doctor, I’m not always great at clearly and confidently getting my point across. My tone is probably too soft. And if the doctor’s swamped, they might just forget what I said. I’ve definitely missed a few chances because of that.” (Pharmacist B)
	**Training programs**	
	(-) The pharmacy lacks sufficient teaching capacity to provide training tailored to individual pharmacists’ needs	“If a new pharmacist hits a snag while doing admission MedRec, it would be much better if they had more than just one senior to ask. If there were more people, they could turn to for help, I think everyone would learn a lot faster.” (Pharmacist C)
	(+) Pharmacists need more hands-on practice	“I feel like admission MedRec is something you really learn by doing. A lot of the time, we’re not that confident because we haven’t had enough practice. If we got more chances to actually try it out, we’d probably use our time better, and it would make a bigger difference in the long run.” (Pharmacist M)
	(+) The pharmacy offers an admission MedRec course	“I took that admission MedRec course. Before that class, I honestly had no idea that admission MedRec was even something we could do.” (Pharmacist L)
**Physical environment**	**Hardware infrastructure**	
	(-) Low-quality keyboards and mice in the pharmacy interrupt workflow and focus	“Some of the keyboards or mice are really difficult to use. It just makes you not want to look up patient info with them. It gets annoying and kind of ruins your mood while working.” (Pharmacist G)
	(-) Poor internet connectivity hinders service delivery	“I think the Wi-Fi in the wards is pretty bad. Right when you need to look something up, maybe a picture of a med or a patient’s info to show them, you just can’t get online.” (Pharmacist A)
	**Information systems**	
	(+) Hospital electronic systems could be developed to integrate admission MedRec functionality	“I feel like admission MedRec should be built right into the hospital system—like, there should be a page you can click on that automatically pulls in the patient’s med history, and you can fill out and save the service note all in one spot.” (Pharmacist L)
	(+) Hospital electronic systems could be improved to flag patients who have not received admission MedRec	“Right now, there’s no easy way to tell from the main ward page which patients were just admitted and still need admission MedRec. You have to click into each chart one by one. Honestly, we usually don’t have time for that, so it’s not something you’d naturally think to do.” (Pharmacist E)
	(+) Hospital electronic systems could be improved to speed up the process of writing service notes	“I feel like the service note template for pharmacists could be much easier to use. Right now, it pulls in some basic patient info, which helps, so we don’t have to type everything out—but it still can’t bring in the patient’s NHI med records. We used to just read them and copy stuff by hand, which was such a hassle. If we’re expected to write these notes, the system should really make it much more straightforward.” (Pharmacist O)
	(-) Pharmacists are unable to access patients’ medication history in a timely manner	“Usually when a patient is first admitted, we can’t see their NHI med history right away. I usually wait until I can check their cloud records before doing anything. A lot of times, meds are already prescribed, but there’s no admission note yet, so we don’t even know what’s going on or what the main diagnosis is. That makes it hard to figure out which meds should be continued or paused when performing admission MedRec” (Pharmacist C)
**Social environment**	**Work situation**	
	(-) Pharmacists lack dedicated time to fully carry out admission MedRec or are frequently interrupted	“We’ve got calls coming in from the wards, need to check the med carts, and handle all kinds of stuff with the automated dispensing cabinets. A lot of that eats up time, and sometimes you’re in the middle of reviewing something when a phone call cuts you off. It really slows things down, and doing admission MedRec for every single patient just isn’t feasible.” (Pharmacist H)
	(-) The pharmacy’s workflow does not support the execution of admission MedRec	“When things get busy or there are a lot of discharges, just dealing with all the administrative tasks can take up the whole day. By the time you’re done, it’s already the end of your shift, so if you want to check on patients or do admission MedRec, you’re basically using your personal time.” (Pharmacist G)
	(-) Pharmacists are overwhelmed by heavy workloads	“Sometimes there are just too many patients, and I can’t get to all of them in one day. There’s no way to finish admission MedRec for everyone when the workload is that heavy.” (Pharmacist K)
	**Institutional norms**	
	(-) The pharmacy lacks a clear internal consensus on the purpose of admission MedRec	“I’m not really sure what the main goal of the admission MedRec project is. When we’re doing it, what exactly are we trying to achieve? If there were a clear purpose, we’d know who is supposed to benefit from it. But right now, I don’t really have a clear picture.” (Pharmacist C)
	(-) The pharmacy lacks a culture or environment that supports fully carrying out admission MedRec	“I think it’s just that no one’s really doing it, so you don’t even realize it’s something you can or should be doing. You don’t feel like there’s a reason to spend extra time writing up the admission MedRec process. Writing the note takes time, and if the people before you never did it, you kind of just assume it’s not part of your job either.” (Pharmacist A)

(+): facilitators, (-): barriers

### Predisposing factors

#### Perceptions of admission MedRec

Most pharmacists demonstrated a clear understanding of the definition and procedures involved in admission MedRec and held generally positive attitudes toward it. Many recognized it as an effective approach to enhancing medication safety by reducing discrepancies and improving medication adherence. Some also noted that performing admission MedRec enhanced their understanding of patients’ medication regimens and contributed to their professional development and job satisfaction.

#### Perceived value of admission MedRec

Despite positive perceptions, pharmacists sometimes questioned the necessity and prioritization of admission MedRec in daily workflow. The process was frequently viewed as time-consuming and secondary to other clinical duties. In particular, documentation, a crucial but labor-intensive step, was often omitted. Several pharmacists doubted whether their documentation was reviewed or used by other healthcare professionals, which reduced its perceived value and, in turn, their motivation to complete it.

### Reinforcing factors

#### Performance feedback

Uncertainty about how admission MedRec contributions were reflected in performance assessments reduced motivation among pharmacists. Although the hospital required electronic signatures as part of the documentation process, this step was often missed due to its misalignment with routine workflow. Fear of potential penalties for missing signatures frequently outweighed any perceived benefits of documentation. Some pharmacists also criticized the performance evaluation system for emphasizing quantifiable metrics over actual effort, which left them feeling that their contributions were undervalued.

#### Community feedback

Pharmacists reported limited feedback from both patients and other healthcare professionals regarding admission MedRec activities. Many observed that their colleagues and patients were either unaware of the service or unclear about the pharmacist’s role in it. As a result, the service often went unrecognized. Without external acknowledgment, pharmacists tended to rely on internal motivation, such as a sense of personal or professional accomplishment, to justify their continued engagement.

### Enabling factors

#### Professional competence

Participants identified several core competencies necessary for effective admission MedRec, including medication knowledge, information gathering, clinical documentation, and communication skills. While pharmacists generally felt well-trained in medication knowledge, many reported insufficient training in documentation and communication. The lack of structured instruction in these areas led to lower confidence, increased time burdens, and reduced efficiency. Information-gathering and communication were also described as common challenges. Difficulties in collecting patient data and limited communication training also hindered effective interprofessional collaboration and patient counseling.

#### Training programs

Although existing training programs provide a basic instruction to admission MedRec, many participants felt these offerings lacked depth and failed to adequately prepare pharmacists for real-world implementation. Several advocated for more personalized and skill-based training that could be tailored to individual learning needs. In particularly, structured, hands-on experience in clinical documentation and patient communication was highlighted as essential. The current mentorship system was also seen as insufficient, with some junior pharmacists noting that mentors lacked the awareness or skills to provide meaningful support. This gap may leave junior pharmacists less supported, less confident, and less efficient in performing admission MedRec.

### Physical environment

#### Hardware infrastructure and information systems

While participants had no major concerns about the availability of hardware, many pointed to deficiencies in systems integration and functionality that hinder admission MedRec documentation. Current digital platforms provide limited support for entering admission MedRec notes, creating a substantial time burden. To improve workflow and motivation, pharmacists proposed systems enhancements, such as integrating medication histories, generating alerts for pending tasks, and allowing comparison of medication records across care settings and points. These improvements were seen as crucial for making admission MedRec more efficient and sustainable.

### Social environment

#### Work situation

Frequent interruptions and a lack of dedicated time for admission MedRec were common challenges. Pharmacists reported that admission MedRec was not formally embedded into their routine responsibilities and often had to be performed alongside other clinical tasks. Given the demanding and unpredictable nature of their workloads, finding time for admission MedRec was difficult and often deprioritized.

#### Institutional norms

A lack of consensus within the pharmacy department regarding the core objective of admission MedRec contributed to inconsistent practices. Some pharmacists believed the primary goal was to identify medication discrepancies, while others emphasized comprehensive documentation. This ambiguity contributed to low documentation rates. Participants also highlighted that the prevailing group climate strongly influences individual behavior. When admission MedRec was valued and actively promoted within the team, through shared norms, collaboration, and leadership support, pharmacists felt more motivated and were more likely to engage consistently with the service.

## Discussion

This study explored the factors that influence hospital pharmacists’ engagement in admission MedRec. Although admission MedRec is widely recognized as a standard component of patient safety initiatives across healthcare settings, it is still a complex and resource-intensive service that poses considerable implementation challenges. To systematically identify the determinants affecting admission MedRec delivery, we used the PRECEDE-PROCEED model as the guiding framework for both the development of the interview guide and the subsequent data analysis. Findings from this study will serve as the foundation for a subsequent quantitative investigation assessing the relative importance of each identified factor and will also support the development of targeted implementation strategies (e.g. reducing barriers and enhancing facilitators). This stepwise approach aligns with current implementation science frameworks, which emphasize tailoring strategies to specific contexts to effectively address setting-dependent barriers [27, 28]. In this regard, the PRECEDE-PROCEED model corresponds well to implementation processes by offering a structured pathway from contextual assessment to strategy design and evaluation. While this study focused on the assessment phase, the findings lay the groundwork for designing and evaluating future implementation strategies. Future efforts should build on these findings to design tailored and context-specific interventions that address identified barriers and leverage facilitators, using the PRECEDE-PROCEED framework as a roadmap [29].

A recurring theme in participants’ narratives was the influence of time-related constraints. Pharmacists frequently described admission MedRec as highly time-consuming, consistent with existing literature that identifies time pressure as a widespread barrier to implementing clinical pharmacy services [30–33]. Based on our observations, performing admission MedRec in our hospital currently requires approximately 15 to 30 min per patient, and other studies have reported similar time demands [8, 34]. These findings underscore the critical importance of time as both a limited and essential resource in hospital systems. When new responsibilities such as admission MedRec are introduced without adequate resource support, frontline pharmacists often perceive them as additional burdens. Inadequate time allocation can therefore undermine both the motivation and capacity of healthcare professionals to engage meaningfully in such initiatives. To address this, hospitals should allocate protected time for pharmacists to conduct admission MedRec and consider adjusting staffing or workflow to ensure sufficient time is available for high-quality execution [35].

Notably, pharmacists did not always express time constraints directly. Instead, challenges were often expressed indirectly through related structural and organizational issues. At the physical environment level, participants cited inefficiencies in information systems, which require repetitive data retrieval and increase documentation time. At the social environment level, some pointed out that admission MedRec had not been integrated into routine workflows, making it difficult to prioritize within the existing time schedule. These insights indicate organizational and structural barriers that extend beyond individual time management and reflect broader issues in system design and organizational support. Streamlining information systems and integrating admission MedRec into standard workflows can reduce structural barriers, making the process more efficient and easier to prioritize [36].

In response to these challenges, several pharmacists placed high expectations for technological solutions, such as automation and streamlined process, to ease their workload demands and make admission MedRec more feasible. However, prior research has shown that technology alone has not consistently improved admission MedRec implementation rates [37]. This suggests that while technological enhancements are helpful, they are insufficient to overcome the systematic constraints pharmacists face. Combining technological improvements with workflow redesign, on-the-job training, and organizational support can ensure that digital tools effectively enhance admission MedRec delivery rather than serve as standalone solutions [38, 39].

Our findings further indicate that, in addition to time pressure, a key barrier is the relative deprioritization of admission MedRec in favor of other pharmaceutical services perceived as more urgent or rewarding. This suggests that the issue is not solely logistical but also cognitive and motivational. Pharmacists’ decisions. about which services to prioritize are influenced by perceived value, expected outcomes, and available feedback. Therefore, implementation strategies must go beyond logistical fixes to directly address how pharmacists perceive and prioritize admission MedRec within the broader context of their professional responsibilities [40]. This perspective is supported by research showing that healthcare professionals’ beliefs and commitment to an intervention significantly affect its uptake and sustainability [41]. Interventions should focus on reinforcing the perceived value of admission MedRec through education, clear communication of its impact, and recognition mechanisms to enhance pharmacists’ motivation and prioritization [42].

From this perspective, the Diffusion of Innovations Theory offers a useful lens for understanding these dynamics [43]. Innovation spreads more effectively when early adopters, those who embrace new practices ahead of their peers, are supported and visible within the organization. These individuals can serve as role models, shaping norms and encouraging broader adoption through positive peer influence. However, our findings indicate that admission MedRec remains in the early stages of diffusion within the pharmacy department. A lack of shared understanding regarding the goals of admission MedRec reflects a fragmented organizational culture. While some pharmacists may act as early adopters, their efforts have not yet translated into sustained momentum. Without clear, top-down directives and institutional support, early adopters alone are unlikely to drive lasting change. To promote adoption and normalization of admission MedRec, formal support for early adopters, leadership endorsement, and clear communication of objectives are essential [44].

Moreover, even highly effective services such as admission MedRec may struggle to maintain long-term engagement without meaningful feedback. Most participants reported limited acknowledgment following the execution of admission MedRec, whether in the form of peer recognition, feedback from other healthcare professionals or patients, or personal fulfillment. The lack of feedback diminishes motivation and may contribute to the service being relegated to a lower priority in daily practice. To ensure the success and sustainability of future implementation strategies, it is crucial to establish organizational mechanisms that provide timely, constructive, and visible feedback [45]. Implementing structured feedback mechanisms, including peer recognition, supervisor appraisal, and patient acknowledgment, can reinforce engagement, enhance the perceived value, and support integration of admission MedRec into routine clinical workflows [46].

### Limitation

This study has some limitations. First, the relatively small sample from a single institution may restrict the broader applicability of the findings. However, the strength of this study lies in its rich and context-specific insights into the implementation landscape. While localized, the findings may offer valuable lessons for other healthcare institutions, which could apply the PRECEDE-PROCEED model to explore the unique contextual factors influencing admission MedRec in their own settings. Second, participants’ responses may have been subjected to social desirability bias [47]. Nevertheless, the interviews were conducted by researchers familiar with the study setting and trained in qualitative methods. This dual combination enabled the use of reflexive interviewing techniques to elicit deeper insights, including perspectives that participants may not consciously recognized, thus enhancing the interpretive validity.

## Conclusion

This study identified key contextual barriers to pharmacist-led admission MedRec, including time pressure, ambiguous task prioritization, and insufficient feedback mechanisms. Although pharmacists generally recognized the value and effectiveness of admission MedRec, competing responsibilities and limited institutional and cultural support caused its deprioritization in daily practice. Using the PRECEDE-PROCEED model as a guiding framework, the study systematically explored factors influencing implementation across individual and environmental levels. These findings provide practical insights for developing targeted implementation strategies to support the sustained and effective integration of admission MedRec in hospital practice.

## Data Availability

The data that support the findings of this study are only available on reasonable request from the corresponding author. The data generated and analyzed in this study are not publicly available because they may contain private information. Furthermore, informed consent ensured that the data would only be used for this study.

## Declarations

### Disclose of the use of generative artificial intelligence (AI)

The authors used generative AI (ChatGPT 4o) to assist with translating texts and language polish, ensuring the research findings are expressed in a more fluent language.

## Abbreviations

MedRec: Medication reconciliation
NHI: National Health Insurance
